# Long-term exposure to PM_2.5_ and NO_2_ and risk of myopia in Chinese school-aged children: a cross-sectional study

**DOI:** 10.1186/s12886-025-04587-7

**Published:** 2026-01-29

**Authors:** Keke Liu, Huijuan Luo, Boran E, Huining Kuang, Chenyu Zhang, Xin Guo

**Affiliations:** 1 School of Public Health, Capital Medical University, No.10, West Toujiao, Right Anmenwai, Fengtai District, Beijing, 100069 People’s Republic of China; 2 School Health Center, Beijing Center for Disease Prevention and Control, No. 16, Hepingli Middle Street, Dongcheng District, Beijing, 100013 People’s Republic of China; 3 Chinese Center for Disease Control and Prevention, Beijing, People’s Republic of China

**Keywords:** Particulate matter, Nitrogen dioxide, Children, Cross-sectional study, Myopia

## Abstract

**Objective:**

To explore the link between long-term exposure to fine particulate matter (PM_2.5_) and nitrogen dioxide (NO_2_) and myopia prevalence in Chinese school-aged children.

**Methods:**

Conducted from September 2023 to June 2024, this cross-sectional study included 23,983 school-aged children from six cities across China. Myopia was defined using non-cycloplegic refraction (SE < -0.50 D) combined with visual acuity testing. Three-year average concentrations of pollutants were sourced from the China High Air Pollutants (CHAP) dataset. We employed adjusted mixed-effects models to evaluate the relationship between exposure to air pollutants and the risk of myopia.

**Results:**

In adjusted linear models, each interquartile range (IQR) increase in long-term PM_2.5_ exposure was associated with significantly higher odds of myopia (odds ratio [OR] = 1.63; 95% confidence interval [CI]: 1.14–2.33). However, non-linear modeling identified a pronounced departure from linearity (*P* for non-linearity < 0.001), characterized by a steep risk increase at lower concentrations followed by a plateau at higher levels. Consistent with this pattern, categorical analyses showed that children in PM_2.5_ exposure quartiles Q2-Q4 had substantially elevated odds of myopia compared with Q1 (OR range: 3.30–3.59).For NO2, the per-IQR association was not statistically significant (OR = 0.96; 95% CI: 0.84–1.09), yet exposure quartiles Q2-Q4 were all associated with significantly increased myopia risk relative to Q1 (OR range: 1.30–1.58). Subgroup analyses suggested variability across sex, grade level, and parental education, but no consistent pattern was observed. Results were robust after adjusting for ozone and using alternative exposure windows.

**Conclusion:**

Our results provide clear evidence that ambient PM_2.5_ is an important environmental risk factor for childhood myopia, with a dose-response pattern indicating heightened vulnerability at commonly encountered exposure levels. The consistent risk associated with higher NO_2_ exposure further supports the role of air pollution. Mitigating myopia burden may therefore require integrating air quality criteria into public health strategies for children’s visual health.

**Supplementary Information:**

The online version contains supplementary material available at 10.1186/s12886-025-04587-7.

## Introduction

Myopia is one of the most prevalent visual disorders among children and adolescents worldwide, particularly in Asia [1]. At present, approximately 2 billion individuals (constituting 28.3% of the world’s population) have been identified with myopia, and it is projected that by 2050, the number of people with myopia will increase to 4.76 billion (49.8% of the global population) [2, 3]. In China, the overall myopia prevalence rate among Chinese children and adolescents reached 51.9% in 2022, with a myopia rate of 36.7% among primary school students, 71.4% among junior high school students, and 81.2% among senior high school students [4]. Moreover, early-onset myopia often progresses to high myopia, which significantly increases the risk of severe complications such as retinal detachment, glaucoma, and macular degeneration [5]. The rapid increase in myopia prevalence over the past decades cannot be explained solely by genetic factors, highlighting the crucial role of environmental and behavioral influences.

Mounting evidence suggests that myopia development results from complex interactions between genetic susceptibility and environmental exposures, with the latter playing a predominant role. In recent years, the issue of atmospheric pollution has garnered growing concern as an alterable environmental determinant of health [6–8]. Both epidemiological and experimental studies indicate that chronic exposure to air pollutants – particularly fine particulate matter (PM_2.5_) and gaseous compounds like nitrogen dioxide (NO₂) – may promote axial elongation and myopia progression through oxidative stress and inflammatory pathways, potentially inducing ocular surface irritation, tear film instability, conjunctivitis, and even retinal damage [9–11]. Notably, the presence of black carbon particles in ambient PM on the human eye surface provides concrete evidence of PM’s direct impact on eye health and its potential risks [12].

Despite these plausible mechanisms, epidemiologic evidence linking chronic exposure to PM_2.5_ and NO_2_ with childhood myopia remains scarce and inconsistent. Previous studies have been limited by small sample sizes, single-city designs, or coarse pollution data. Moreover, few have accounted for behavioral confounders such as outdoor activity, screen time, and parental myopia. Therefore, large-scale, multi-regional studies using high-resolution exposure estimates are urgently needed to clarify these associations. In this study, we investigated the relationship between long-term exposure to PM_2.5_ and NO_2_ and the risk of myopia among school-aged children from three provinces in China. We hypothesized that higher chronic exposure to these pollutants would be associated with an increased likelihood of myopia, independent of demographic, behavioral, and meteorological factors.

## Methodology

### Study population

A cross-sectional survey was conducted in China from September 2023 to June 2024 using a multi-stage random sampling strategy. After randomly selecting Guangdong, Hunan, and Shanxi provinces, we chose two representative cities from each via systematic sampling. We further employed a cluster sampling approach in which schools were treated as the primary sampling units; within each selected school, all eligible classes or students were invited to participate. The inclusion criteria were: (1) children aged 5–13 years; (2) currently enrolled in local kindergartens or primary schools; and (3) guardians provided signed informed consent. Exclusion criteria were based on: (1) congenital eye diseases or a history of eye surgery; (2) systemic diseases affecting vision; and (3) incomplete or missing responses in key questionnaire items. Demographic information, lifestyle patterns, and visual health data were obtained using standardized, self-administered survey instruments. A total of 30,785 eligible children were recruited. After excluding children with missing vision data (*n* = 4,533) and incomplete demographic information (*n* = 2,269), 23,983 children remained with complete baseline information. All remaining children provided valid questionnaires (response rate: 77.9%) and were included in the final analysis (Fig. S1).

All participants, or their legal guardians if they were minors, provided informed consent. The consent was obtained in written form and was documented and witnessed by a member of the research team. The study protocol was officially endorsed by the Institutional Review Board of the Beijing Municipal Center for Disease Prevention and Control (IRB approval number 24, 2022), and all research activities adhered to the ethical guidelines outlined in the Declaration of Helsinki.

### Myopia screening

Ophthalmic examinations were conducted by licensed medical professionals at designated institutions. Following a slit-lamp examination to rule out contraindications, refractive error was measured using an automated refractor without cycloplegia. Both refractions for each subject were performed by the same optometrist. The spherical equivalent (SE) was calculated as the spherical power plus half of the cylindrical power. Myopia was defined as naked eye visual acuity < 5.0 and non-cycloplegic computerized optometry SE <-0.50 D [13].

### Estimation of environmental exposures

Daily concentrations of ambient NO_2_ and PM_2.5_ were sourced from the China High Air Pollutants (CHAP) dataset [14, 15]. This artificial intelligence-enhanced dataset was generated through the fusion of multi-source inputs, including ground-based monitoring, satellite remote sensing outputs, atmospheric reanalysis, and numerical model simulations, with explicit consideration of spatiotemporal variations in pollution levels. The 10-fold cross-validation demonstrated high predictive accuracy, with R² values of 0.92 for PM_2.5_ and 0.93 for NO_2_. For each participant, the annual average concentrations were assigned based on the geocoded school address, meaning that exposure estimates reflected school-level rather than individual-level variation. We then computed 3-year mean concentrations (2021–2023) preceding the survey to represent chronic exposure levels. This approach is consistent with previous epidemiological studies of long-term air pollution and child health.

### Covariates

Covariates were collected using a structured questionnaire adapted for the present survey by the Beijing Municipal Center for Disease Prevention and Control, based on the standardized Student Common Diseases and Health Influencing Factors Surveillance Questionnaire used in school-based health monitoring in China. To ensure data reliability, the internal consistency of the survey sections used in our analysis was assessed, yielding a Cronbach’ s alpha coefficient of 0.81. For children aged < 10 years, questionnaires were completed by parents or guardians; for older children, they were self-administered under teacher supervision. Demographic information included educational stage (kindergarten, lower primary school [grades 1–3], or upper primary school [grades 4–6]), sex (boys or girls), maternal education and parental education levels (junior high school or below, high school/vocation, associate degree, bachelor’s degree, or graduate degree), parental myopia (paternal myopia only, maternal myopia only, biparental myopia, no parental myopia, or parental myopia unknown), and preterm birth (< 37 weeks; yes, no, or unknown). Behavioral assessments covered daily outdoor activity time, television viewing duration, and after-school homework hours. All variables were derived from questions with clear operational definitions to ensure data quality and consistency. The full English version of the adapted survey instrument is available from the corresponding author upon reasonable request for academic verification purposes.

Gridded meteorological variables, specifically ambient temperature and relative humidity at a 9 × 9 km spatial resolution, were sourced from the fifth-generation European Reanalysis-Land (ERA5-Land) reanalysis product [16]. Daily ambient ozone (O_3_) concentrations were acquired from the CHAP dataset at a finer 1 × 1 km resolution [17]. Three-year averaged levels of these variables—temperature, relative humidity, and O_3_ —were incorporated into the analyses as adjusting covariates.

### Statistical analysis

Descriptive statistics were presented as means ± standard deviation (SD) for continuous variables and as counts (percentages) for categorical variables. Associations between pollutant exposure and myopia were assessed using generalized linear mixed models (GLMMs) with a logit link, incorporating a random intercept for province (and, in sensitivity analyses, a random intercept for school) to account for within-province clustering. Because exposure was assigned at the school level, the data inherently feature a school-city-province hierarchy. Although we explored models additionally incorporating school-level or city-level random effects, model convergence and overparameterization issues prevented their use in the primary analysis; therefore, the province-level random intercept was retained. All models were adjusted for age, sex, grade, parental education, parental myopia, preterm birth, outdoor time, after-school study time, TV viewing time, and meteorological covariates (mean temperature and relative humidity). O_3_ was further included in two-pollutant models to evaluate co-pollutant confounding.

Pollutant concentrations were analyzed both as continuous variables (per interquartile range [IQR] increase) and as quartiles (Q1–Q4) to assess dose–response trends. Median values within quartiles were modeled as continuous variables to test for linear trends. To explore non-linearity, we applied natural cubic splines with 3 degrees of freedom and used likelihood ratio tests to compare against linear models.

Finally, several sensitivity analyses were performed to evaluate the robustness of results: (1) inclusion of O_3_ in two-pollutant models; (2) extending exposure assessment to 5-year averages (2019–2023). We further conducted stratified analyses by sex, educational stage (lower vs. upper primary), and parental education level to assess potential effect modification. Interaction terms between pollutant exposure and stratifying variables were tested to evaluate statistical significance of heterogeneity. All statistical analyses were performed utilizing the R programming language (specifically version 4.4.2). A *P*-value of below 0.05 on both sides was considered to indicate statistical significance.

## Results

### Overview of participant traits and pollution exposure metrics

Table 1 outlines participant demographics. The final analytical sample comprised 23,983 participants, among whom 3,852 (16.1%) were diagnosed with myopia. The average age of the cohort was 7.2 years (SD = 1.7), with 12,531 boys (52.2%) and 11,452 girls (47.8%). Compared with children without myopia, children with myopia were more likely to be in Upper Primary School (Grade 4–6) (48.2% vs. 10.6%), have a lower level of maternal education (below college: 53.7% vs. 47.8%), have a lower level of paternal education (below college: 55.1% vs. 50.6%), have a higher degree of parental myopia (At least one parent has myopia: 64.7% vs. 47.8%), be preterm (8.6% vs. 7.8%), engage in less than 2 h of daily outdoor activity (53.5% vs. 50.2%), daily TV watching time for 4 h or more (3.6% vs. 2.7%), and spend 2 h or more on daily after-school learning time (22.2% vs. 14%). In addition, from 2021 to 2023, the average concentrations of PM_2.5_, NO_2_, and O_3_ were 33.3 (± 11.3) µg/m³, 25.1 (± 6.6) µg/m³, and 104.7 (± 8.6) µg/m³, respectively (Table 2).

**Table 1:** Baseline characteristics of study participants by myopia status

Characteristics	Study participants (*N* = 23983)	No-Myopia (*N* = 20131)	Myopia (*N* = 3852)	*P* Value
**Age**	7.2 (1.7)	6.9 (1.5)	8.8(1.8)	*P* < 0.001
**Grade**,** n (%)**				*P* < 0.001
Kindergarten	6159 (25.7)	5944 (29.5)	215 (5.6)	
Lower Primary School (Grade 1–3)	13,825 (57.6)	12,045 (59.8)	1780 (46.2)	
Upper Primary School (Grade 4–6)	3999 (16.7)	2142 (10.6)	1857 (48.2)	
**Sex**,** n (%)**				
Boys	12,531 (52.2)	10,558 (52.4)	1973 (51.2)	*P* < 0.001
Girls	11,452 (47.8)	9573(47.6)	1879 (48.8)	
**Maternal education**,** n (%)**				*P* < 0.001
Junior high or below	4495 (18.7)	3642 (18.1)	853 (22.1)	
High school/vocational	7194 (30)	5978 (29.7)	1216 (31.6)	
Associate degree	5974 (24.9)	5049 (25.1)	925 (24.0)	
Bachelor’s degree	5635 (23.5)	4867 (24.2)	768 (19.9)	
Graduate degree	685 (2.9)	595 (3.0)	90 (2.3)	
**Paternal education**,** n (%)**				*P* < 0.001
Junior high or below	4820 (20.1)	3951 (19.6)	869 (22.6)	
High school/vocational	7497 (31.3)	6247 (31)	1250 (32.5)	
Associate degree	5464 (22.8)	4588 (22.8)	876 (22.7)	
Bachelor’s degree	5357 (22.3)	4614 (22.9)	743 (19.3)	
Graduate degree	845 (3.5)	731(3.6)	114 (3)	
**Parental myopia**,** n (%)**				*P* < 0.001
Paternal myopia only	9225 (38.5)	2848 (14.1)	589 (15.3)	
Maternal myopia only	3437 (14.3)	4656 (23.1)	921 (23.9)	
Biparental myopia	5577 (23.3)	3758 (18.7)	984 (25.5)	
No parental myopia	4742 (19.8)	8049 (40)	1176 (30.5)	
Parental myopia unknown	1002 (4.2)	820 (4.1)	182 (4.7)	
**Preterm birth(<37weeks)**,** n (%)**				0.015
Yes	1908 (8)	1578 (7.8)	330(8.6)	
No	21,512 (89.7)	18,101 (89.9)	3411 (88.6)	
unknown	563 (2.3)	452 (2.2)	111(2.9)	
**Daily outdoor activity time**,** n (%)**				*P* < 0.001
< 2 h	12,172 (50.8)	10,112 (50.2)	2060 (53.5)	
≥ 2 h	9039 (37.7)	7718 (38.3)	1321 (34.3)	
unclear	2772 (11.6)	2301 (11.4)	471 (12.2)	
**Daily TV watching time**,** n (%)**				*P* < 0.001
<2 h	20,387 (85.0)	17,212 (85.5)	3175 (82.4)	
2–4 h	2920 (12.2)	2380 (11.8)	540 (14)	
≥ 4 h	676 (2.8)	539 (2.7)	137 (3.6)	
**Daily after-school learning time**,** n (%)**				*P* < 0.001
< 2 h	19,756 (82.4)	16,885 (83.9)	2871 (74.5)	
≥ 2 h	3681 (15.3)	2824 (14)	857 (22.2)	
unclear	546 (2.3)	422 (2.1)	124 (3.2)	
**Left eye SE**	0.6 (13.7)	1.0 (15.0)	-1.7 (1.5)	*P* < 0.001
**Right eye SE**	0.5 (9.8)	0.9 (10.6)	-1.8 (1.5)	*P* < 0.001

Abbreviations: PM_2.5_, particles with aerodynamic diameter ≤ 2.5 μm; NO_2_, nitrogen dioxide; O_3_, ozone; SD, standard error; SE, spherical equivalent

**Table 2:** Summary statistics of individual exposure

Exposure ^*^	Mean (SD)	Percentile				
		min	25	50	75	max
PM_2.5_, µg/m^3^	33.25 (11.29)	18.46	22.01	34.49	40.48	54.71
NO_2_, µg/m^3^	25.06 (6.64)	16.09	18.34	25.83	28.41	38.6
O_3_, µg/m^3^	104.70(8.56)	92.60	98.51	102.33	111.27	121.76
Temperature, °C	8.74 (5.19)	-2.92	6.45	6.81	14.35	14.92
Relative humidity, %	53.25 (9.61)	33.98	48.0	54.07	63.42	64.79

Abbreviations: Individual exposure was calculated as the average value over the three years preceding the survey. Abbreviations: SD, standard error; PM_2.5_, particulate matter with an aerodynamic diameter ≤ 2.5 μm; NO_2_, nitrogen dioxide; O_3_, ozone; µg/m^3^,micrograms per cubic meter

### Effects of air pollutants on myopia risk

In the fully adjusted models, each interquartile range (IQR) increase in PM_2.5_ was associated with a significantly higher likelihood of myopia (OR [odds ratio] = 1.63; 95% CI: 1.14–2.33). In contrast, the linear IQR-based estimate for NO_2_ showed no significant association (OR = 0.96; 95% CI: 0.84–1.09). However, the quartile analyses revealed clearer patterns for both pollutants. For PM_2.5_, children in exposure quartiles Q2 - Q4 all showed markedly elevated odds of myopia relative to Q1, with ORs of 3.59 (95% CI: 2.95–4.38), 3.30 (95% CI: 2.52–4.32), and 3.49 (95% CI: 2.51–4.84), respectively. Similarly, NO_2_ exposure demonstrated significantly higher risks in Q2–Q4 compared with Q1 (ORs: 1.58, 1.34, and 1.30), despite the null linear estimate. This pattern indicates a non-linear or threshold-like dose-response, where risk increases occur across specific exposure intervals rather than uniformly across the distribution—an effect not captured by the IQR-based linear model (Table 3).

**Table 3:** Associations of PM_2.5_ and NO_2_ exposure with myopia

Air Pollutants	Myopia	Left eye SE	Right eye SE
	ORs and 95% CIs	β and 95% CIs	β and 95% CIs
**PM2.5, µg**			
per IQR increase	**1.63 (1.14**,** 2.33)**	0.18 (-1.20,1.56)	0.04 (-0.95, 1.02)
Quartile 1 (18.46–22.01)	Ref		
Quartile 2 (22.01–34.49)	**3.59 (2.95**,** 4.38)**	-0.62 (-1.39, 0.16)	**-0.58 (-1.15**,** -0.01)**
Quartile 3 (34.49–40.48)	**3.30 (2.52**,** 4.32)**	-0.48 (-1.51, 0.55)	-0.37 (-1.12, 0.39)
Quartile 4 (40.48–54.71)	**3.49 (2.51**,** 4.84)**	-0.62 (-1.98, 0.74)	-0.59 (-1.55, 0.38)
**NO**_**2**_, **µg**			
per IQR increase	0.96 (0.84,1.09)	-0.09 (-0.45,0.28)	-0.09(-0.36,0.19)
Quartile 1 (16.09–18.34)	Ref		
Quartile 2 (18.34–25.83)	**1.58 (1.38**,** 1.82)**	-0.18 (-0.76, 0.40)	**-0.49 (-0.90**,** -0.07)**
Quartile 3 (25.83–28.41)	**1.34 (1.10**,** 1.62)**	-0.20 (-0.97, 0.57)	-0.29 (-0.84, 0.26)
Quartile 4 (28.41–38.60)	**1.30 (1.09**,** 1.54)**	-0.28 (-0.94, 0.38)	-0.44 (-0.91, 0.03)

Abbreviations: The analysis was adjusted for various demographic and behavioral variables, including age, sex, maternal education, paternal education, parental myopia, preterm birth, daily outdoor activity time, daily after-school learning time, and daily TV watching time. Environmental factors, including average temperature and relative humidity from three years before the survey, were modeled using natural cubic splines (3 degrees of freedom each). Abbreviations: PM_2.5_, particles with aerodynamic diameter ≤ 2.5 μm; NO_2_, nitrogen dioxide; IQR, interquartile range; OR, odds ratio; CI, confidence interval; µg, microgram

Regarding spherical equivalent (SE), the right-eye models showed significant negative associations for Q2 PM_2.5_ (*β* = -0.58; 95% CI: -1.15, -0.01) and Q2 NO_2_ (*β* = -0.49; 95% CI: -0.90, -0.07). The corresponding associations for Q3 and Q4 for both pollutants followed similar negative directions but did not reach statistical significance. In contrast, no PM_2.5_ or NO_2_ quartile demonstrated statistically significant associations for left-eye SE. This asymmetry may reflect modest exposure effects relative to measurement variability and small physiological interocular differences (Table 3).

**Fig. 1:**
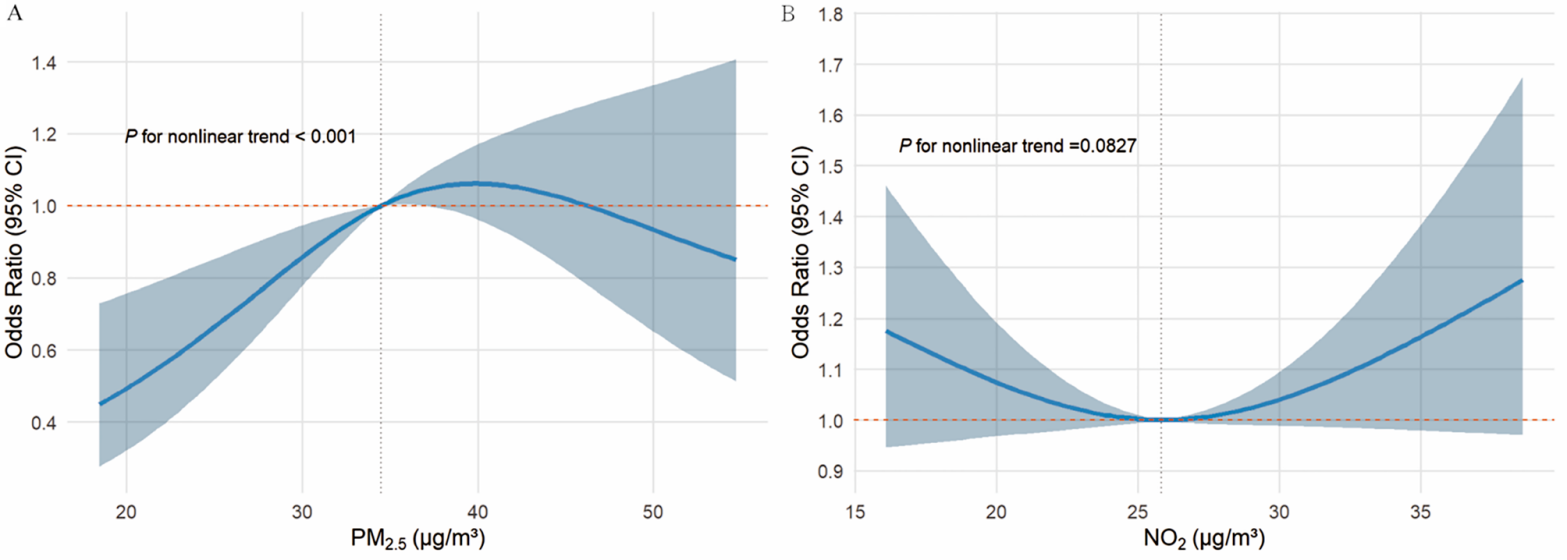
Dose-response associations linking PM_2.5_, NO_2_ exposure to myopia development. The analysis was adjusted for various demographic and behavioral variables, including age, sex, maternal education, paternal education, parental myopia, premature birth, daily outdoor activity time, daily after-school learning time, daily TV watching time. Environmental factors including average temperature and relative humidity from three years before the survey were modeled with natural cubic splines (3 degrees of freedom each). Abbreviations: PM_2.5_, particles with aerodynamic diameter ≤ 2.5 μm; NO_2_, nitrogen dioxide; CI, confidence interval; µg/m3,micrograms per cubic meter

Non-linearity tests further supported these results. For PM_2.5_, we observed a significant departure from linearity (*P* < 0.001), with risk increasing steeply at lower concentrations and plateauing at higher levels. In contrast, NO_2_ did not show a statistically significant non-linear pattern (*P* = 0.0827), despite exhibiting elevated risks in higher quartiles. Collectively, these findings suggest that categorical models more accurately characterize the exposure-response relationship for NO_2_ (Fig. 1).

### Stratified analyses

In stratified analyses, the associations of PM_2.5_ and NO_2_ with myopia showed variability across subgroups defined by sex, grade level, and parental education (Supplementary Tables S1-S2). Among boys, upper primary school students (grades 4–6), and children whose parents had lower educational attainment, the odds ratios across exposure quartiles (Q2-Q4 vs. Q1) generally followed an increasing pattern. However, these trends were not fully consis76tent across all exposure categories, and several interaction tests did not reach statistical significance. Therefore, while some subgroup patterns were observed, they should be interpreted with caution given the multiple comparisons and the variability in effect estimates.

### Sensitivity analysis

Supplementary analyses incorporating O_3_ as an additional covariate yielded consistent effect estimates for the associations of both PM_2.5_ and NO_2_ with myopia (Supplementary Table S3). In addition, we also assessed the associations of PM_2.5_ and NO_2_ exposure for the 5 years before 2024 (Supplementary Table S4).

## Discussion

This large-scale cross-sectional study confirms a significant association between long-term exposure to PM_2.5_ and an increased risk of myopia among Chinese school-aged children. The association was particularly pronounced in boys, older students, and children of parents with lower educational attainment. While the relationship with NO_2_ was weaker in continuous models, a significant trend in the quartile analysis suggests potential threshold or cumulative effects. These findings position ambient air pollution as an emerging environmental risk factor for childhood myopia.

According to the World Health Organization, over 93% of children under the age of 15 globally are exposed to PM_2.5_ levels exceeding health guidelines [18]. Additionally, since children’s respiratory intake per unit of body weight is 1–2 times that of adults, they inhale more air pollutants and are more susceptible to the health hazards brought by air pollutants than adults [19]. The observed associations between PM_2.5_, NO_2_, and myopia in this study are broadly consistent with previous findings. A large retrospective cohort study in Taiwan involving 97,306 children reported that higher PM_2.5_ and NOx concentrations were positively correlated with the risk of myopia [7]. Similarly, a multi-province study in China observed significant relationships between prolonged exposure to PM_1_, PM_2.5_, PM_10_, and NO_2_ and an increased prevalence of visual impairment among school-aged children [8]. Moreover, in a cohort study in Barcelona, Spain, involving 2,727 schoolchildren, Dadvand et al. (2017) found that chronic exposure to PM_2.5_ absorbance (2.3 × 10^−^⁵/m³) and NO_2_ (67.9 µg/m³) was significantly associated with the risk of wearing glasses among school-age children, indirectly reflecting the potential link between pollutant exposure and myopia development [20].

Our stratified analysis suggested potential variation in the associations between air pollution and myopia across certain subgroups, although these findings were not fully consistent across all exposure metrics and interaction tests. Boys, upper primary school students, and children from lower socioeconomic backgrounds, particularly those with less educated parents, showed somewhat stronger associations in several exposure categories, these patterns should be interpreted cautiously due to the lack of consistently significant interaction terms and the possibility of chance findings arising from multiple comparisons. These differences may arise from both behavioral and biological factors. Although outdoor activity is an established protective factor against myopia, boys’ greater outdoor time does not contradict this protective effect [21]. Rather, boys may spend more time outdoors in higher-pollution microenvironments, leading to greater pollutant exposure. Importantly, our regression models have already adjusted for outdoor activity time and near-vision time, indicating that the slightly stronger associations observed in boys likely reflect residual differences in exposure patterns or biological susceptibility rather than differences in outdoor activity itself. Sex-related variations in airway permeability and particle deposition could alter pollutant absorption and subsequent physiological responses [8, 22]. The somewhat stronger associations among older children may primarily reflect longer cumulative pollutant exposure rather than differences in academic burden, although the evidence is not definitive [23, 24]. Because after-school study time (a proxy for academic workload) has been included as a covariate in our models, we cannot attribute the age-related pattern to academic load alone. Therefore, the age-specific pattern may be more plausibly driven by cumulative exposure duration. Meanwhile, air pollution is not confined to the outdoors; infiltration can bring outdoor air pollutants indoors [25]. As children grow older, their cumulative exposure time to pollutants increases, which may potentially exacerbate the potential adverse effects on the eyes and thereby heighten the risk of myopia. Furthermore, children from families with lower educational backgrounds are more likely to live in polluted neighborhoods and may lack awareness or resources for vision protection and air quality management [26]. These combined factors may contribute to increased susceptibility, although the subgroup patterns in our study require cautious interpretation. The potential for residual confounding and the need for more robust statistical approaches, such as controlling for additional variables or using interaction terms more rigorously, should be considered in future studies.

These combined factors could magnify susceptibility to pollution-related ocular effects. The exposure-response patterns observed in this study further indicate nonlinear relationships between pollutant levels and myopia risk. The inverted U-shaped curve for PM_2.5_ suggests a possible saturation or behavioral adaptation effect. Risk increased with pollution up to moderate concentrations, beyond which additional exposure produced minimal incremental change. Although reduced outdoor time during episodes of severe pollution may partially contribute to this plateau, outdoor activity has been included as a covariate in our models. Therefore, the nonlinearity is more likely to reflect biological saturation or physiological mechanisms rather than behavioral differences alone. In contrast, the weak U-shaped curve for NO_2_ may indicate mixed pollution sources or residual confounding related to urbanization, as NO_2_ levels often co-vary with traffic density and socioeconomic conditions. Importantly, these nonlinear trends do not contradict the main hypothesis but rather highlight the complex interplay among behavioral, environmental, and physiological factors in shaping pollution–myopia associations.

Several plausible biological mechanisms may explain how air pollution contributes to myopia development. Air pollutants (such as PM_2.5_ and NO_2_) can directly act on the ocular surface, inducing allergic reactions and enhancing inflammatory responses, thereby increasing the incidence of myopia [27–29]. Chronic systemic inflammation and microvascular dysfunction could also reduce retinal perfusion, contributing to scleral remodeling and axial elongation [10, 30–32]. Indirect pathways primarily involve suppressed dopamine release - a crucial retinal neurotransmitter that regulates ocular development and refraction [33]. Atmospheric pollutants diminish ground-level sunlight through scattering and absorption [34, 35], which diminishes children’s sunlight exposure during outdoor activities, thereby inhibiting dopamine metabolism and promoting myopia development [36]. The critical refractive development window from ages 3–12, with a peak onset at 8–12 years [37], underscores the heightened vulnerability during this period of environmental susceptibility.

This study has several limitations. First, its cross-sectional design precludes causal inference. Second, the use of school addresses as exposure proxies may cause misclassification [38], while unmeasured confounders remain possible. Because exposure was assigned at the school level, some ecological and Berkson-type error is unavoidable and may attenuate the observed associations. Third, our models incorporated province-level random effects but could not include school- or city-level clustering due to data constraints, which may lead to incomplete adjustment for the nested data structure. Finally, the analysis focused only on PM_2.5_ and NO_2_. The subgroup findings should also be interpreted cautiously, as interaction tests were not consistently significant and chance findings due to multiple comparisons cannot be ruled out. Future longitudinal studies incorporating personal exposure monitoring and more comprehensive multilevel modeling are needed to clarify causal pathways and underlying mechanisms.

From a public health perspective, these findings highlight the need to integrate environmental considerations into childhood eye health strategies. Priorities include strengthening vision screening in high-pollution areas, enhancing public awareness of air pollution’s ocular risks, promoting safe outdoor activities, improving indoor ventilation and filtration, and implementing targeted health education for families. Ultimately, controlling ambient air pollution through broader emission-reduction policies could serve as a critical population-level intervention.

## Conclusion

This large-scale study provides evidence that long-term exposure to PM_2.5_ is associated with an increased risk of myopia in Chinese school-aged children, exhibiting a non-linear dose-response relationship characterized by a steep rise in risk at lower exposure levels. For NO_2_, while no linear trend was observed, higher exposure categories were consistently associated with greater risk. These findings position ambient air pollution, particularly PM_2.5_, as a significant environmental risk factor for childhood myopia. Our results underscore the potential child health benefits of stringent air quality management, even within current regulatory limits. Future longitudinal studies with personal exposure assessment are needed to confirm causality and elucidate the underlying biological mechanisms.

## Supplementary Information

Below is the link to the electronic supplementary material.

Supplementary Material 1

## Data Availability

The datasets analyzed during the current study are not publicly available due to confidentiality, but are available from the corresponding author on reasonable request.

## Abbreviations

PM_2.5_: Particularly fine particulate matter
NO_2_: Nitrogen dioxide
O_3_: Ozone
SE: Spherical equivalent
CHAP: China High Air Pollutants
SD: Standard deviation
ERA5-Land: Fifth-generation European Reanalysis-Land
IQR: Interquartile range
OR: Odds ratio
CI: Confidence interval
µg: Microgram
µg/m^3^: Micrograms per cubic meter

## References

[1] Morgan IG, Ohno-Matsui K, Saw S, Myopia. Lancet. 2012;379(9827):1739–48. 22559900 10.1016/S0140-6736(12)60272-4

[2] Baird PN, Saw SM, Lanca C, Guggenheim JA, Smith IE, Zhou X, Matsui KO, Wu PC, Sankaridurg P, Chia A, et al. Myopia. Nat Rev Dis Primers. 2020;6(1):99. 10.1038/s41572-020-00231-4 33328468

[3] Morgan IG, French AN, Ashby RS, Guo X, Ding X, He M, Rose KA. The epidemics of myopia: aetiology and prevention. PROG RETIN EYE RES. 2018;62:134–49. 28951126 10.1016/j.preteyeres.2017.09.004

[4] General Office of the State Council of the People’s Republic of China. Myopia Prevalence Among Chinese Children and Adolescents. In.; 2024.

[5] Haarman A, Enthoven CA, Tideman J, Tedja MS, Verhoeven V, Klaver C. The complications of myopia: A review and Meta-Analysis. Invest Ophthalmol Vis Sci. 2020;61(4):49. 32347918 10.1167/iovs.61.4.49 PMC7401976

[6] Ruan Z, Qian ZM, Guo Y, Zhou J, Yang Y, Acharya BK, Guo S, Zheng Y, Cummings-Vaughn LA, Rigdon SE, et al. Ambient fine particulate matter and Ozone higher than certain thresholds associated with myopia in the elderly aged 50 years and above. ENVIRON RES. 2019;177:108581. 31323395 10.1016/j.envres.2019.108581

[7] Wei CC, Lin HJ, Lim YP, Chen CS, Chang CY, Lin CJ, Chen JJ, Tien PT, Lin CL, Wan L. PM2.5 and nox exposure promote myopia: clinical evidence and experimental proof. Environ Pollut. 2019;254(Pt B):113031. 10.1016/j.envpol.2019.113031 31454569

[8] Yang BY, Guo Y, Zou Z, Gui Z, Bao WW, Hu LW, Chen G, Jing J, Ma J, Li S, et al. Exposure to ambient air pollution and visual impairment in children: A nationwide cross-sectional study in China. J HAZARD MATER. 2021;407:124750. 33341569 10.1016/j.jhazmat.2020.124750

[9] Han Z, Zhao C, Li Y, Xiao M, Yang Y, Zhao Y, Liu C, Liu J, Li P. Ambient air pollution and vision disorder: a systematic review and meta-analysis. Toxics. 2024;12(3). 10.3390/toxics12030209 PMC10975782 38535942

[10] Adar SD, Klein R, Klein BE, Szpiro AA, Cotch MF, Wong TY, O’Neill MS, Shrager S, Barr RG, Siscovick DS, et al. Air pollution and the microvasculature: a cross-sectional assessment of in vivo retinal images in the population-based multi-ethnic study of atherosclerosis (MESA). PLOS MED. 2010;7(11):e1000372. 21152417 10.1371/journal.pmed.1000372 PMC2994677

[11] Jung SJ, Mehta JS, Tong L. Effects of environment pollution on the ocular surface. OCUL SURF. 2018;16(2):198–205. 29510225 10.1016/j.jtos.2018.03.001

[12] Liu L, Lin Y, Min K, Xiao K, Di Y, Peng L, Zhang W, Long Q, Liu Q, Jiang G. Detection of ambient black carbon in conjunctival sac washed fluid reveals the ocular exposure risks of particulate pollution. ENVIRON SCI TECH LET. 2023;10(8):628–34.

[13] National Health Commission of the People’s Republic of China. Specification for screening of refractive error in primary and secondary school students (WS/T 663–2020). In., vol. 2025; 2020.

[14] Wei J, Liu S, Li Z, Liu C, Qin K, Liu X, Pinker RT, Dickerson RR, Lin J, Boersma KF, et al. Ground-Level NO(2) surveillance from space across China for high resolution using interpretable Spatiotemporally weighted artificial intelligence. ENVIRON SCI TECHNOL. 2022;56(14):9988–98. 35767687 10.1021/acs.est.2c03834 PMC9301922

[15] Wei J, Li Z, Cribb M, Huang W, Xue W, Sun L, Guo J, Peng Y, Li J, Lyapustin A, et al. Improved 1 Km resolution PM2.5 estimates across China using enhanced space–time extremely randomized trees. ATMOS CHEM PHYS. 2020;20(6):3273–89.

[16] Muññoz-Sabater J, Dutra E, Agustíí-Panareda A, Albergel C, Arduini G, Balsamo G et al. ERA5-Land: a state-of-the-art global reanalysis dataset for landapplications. Earth Syst Sci Data. 2021; 13(9):4349–83.

[17] Wei J, Li Z, Li K, Dickerson RR, Pinker RT, Wang J, Liu X, Sun L, Xue W, Cribb M. Full-coverage mapping and Spatiotemporal variations of ground-level Ozone (O3) pollution from 2013 to 2020 across China. REMOTE SENS ENVIRON. 2022;270:112775.

[18] World Health Organization. Air pollution and child health: prescribing clean air [EB/0L]. In., vol. Accessed 10 June 2025; 2018.

[19] World Health Organization. Regional Office for Europe & European Centre for Environment and Health. Effects of air pollution on children’s health and development: a review of the evidence. In.: Copenhagen: WHO Regional Office for Europe; 2005.

[20] Dadvand P, Nieuwenhuijsen MJ, Basagana X, Alvarez-Pedrerol M, Dalmau-Bueno A, Cirach M, Rivas I, Brunekreef B, Querol X, Morgan IG, et al. Traffic-related air pollution and spectacles use in schoolchildren. PLoS ONE. 2017;12(4):e167046. 10.1371/journal.pone.0167046 PMC5378327 28369072

[21] Li JH, Zeng HX, Wei J, Wu QZ, Qin SJ, Zeng QG, Zhao B, Dong GH, Shen JC, Zeng XW. Long-term exposure to PM(2.5) and its constituents and visual impairment in schoolchildren: A population-based survey in Guangdong province, China. ENVIRON INT. 2025;195:109270. 39813955 10.1016/j.envint.2025.109270

[22] Clougherty JE. A growing role for gender analysis in air pollution epidemiology. Environ Health Perspect. 2010;118(2):167–76. 20123621 10.1289/ehp.0900994 PMC2831913

[23] Zhang D, Sun B, Wu M, Liu H, Zhou L, Guo L. Prevalence and associated factors of myopia among school students in Shenyang, china: a cross-sectional study. Front Public Health. 2023;11:1239158. 37711238 10.3389/fpubh.2023.1239158 PMC10499391

[24] Hu X, Yuan X, Li H, Gong H, Fu Z, Xie Y, Wang L, Rui D. Trends in myopia prevalence among children and adolescents: a large-scale cross-sectional study in Shihezi, China. BMC Public Health. 2025;25(1):1576. 40296057 10.1186/s12889-025-22790-5 PMC12036207

[25] Fu N, Kim MK, Huang L, Liu J, Chen B, Sharples S. Experimental and numerical analysis of indoor air quality affected by outdoor air particulate levels (PM(1.0), PM(2.5) and PM(10)), room infiltration rate, and occupants’ behaviour. SCI TOTAL ENVIRON. 2022;851(Pt 2):158026. 35973538 10.1016/j.scitotenv.2022.158026

[26] Johnston R, Fowler C, Wilson V, Kelly M. Opportunities for nurses to increase parental health literacy: a discussion paper. Issues Compr Pediatr Nurs. 2015;38(4):266–281. 10.3109/01460862.2015.1074318 26368012

[27] Hong J, Zhong T, Li H, Xu J, Ye X, Mu Z, Lu Y, Mashaghi A, Zhou Y, Tan M, et al. Ambient air pollution, weather changes, and outpatient visits for allergic conjunctivitis: A retrospective registry study. Sci Rep. 2016;6:23858. 27033635 10.1038/srep23858 PMC4817244

[28] Miyazaki D, Fukagawa K, Fukushima A, Fujishima H, Uchio E, Ebihara N, Shoji J, Takamura E, Namba K, Ohashi Y, et al. Air pollution significantly associated with severe ocular allergic inflammatory diseases. Sci Rep. 2019;9(1):18205. 31796815 10.1038/s41598-019-54841-4 PMC6890742

[29] Lasagni VR, Hvozda AA, Janezic NS, Marchini T, Tau J, Martinefski M, Tesone AI, Racca L, Reides CG, Tripodi V, et al. Urban air pollution induces redox imbalance and epithelium hyperplasia in mice cornea. Toxicol Appl Pharmacol. 2019;384:114770. 31628919 10.1016/j.taap.2019.114770

[30] Provost EB, Int PL, Saenen ND, Kicinski M, Louwies T, Vrijens K, De Boever P, Nawrot TS. Recent versus chronic fine particulate air pollution exposure as determinant of the retinal microvasculature in school children. ENVIRON RES. 2017;159:103–10. 28783615 10.1016/j.envres.2017.07.027

[31] Liu Y, Wang L, Xu Y, Pang Z, Mu G. The influence of the choroid on the onset and development of myopia: from perspectives of choroidal thickness and blood flow. ACTA OPHTHALMOL. 2021;99(7):730–8. 33550704 10.1111/aos.14773

[32] Wang T, Li H, Zhang R, Yu Y, Xiao X, Wu C. Evaluation of retinal vascular density and related factors in youth myopia without maculopathy using OCTA. Sci Rep. 2021;11(1):15361. 34321564 10.1038/s41598-021-94909-8 PMC8319333

[33] Zhou X, Pardue MT, Iuvone PM, Qu J. Dopamine signaling and myopia development: what are the key challenges. PROG RETIN EYE RES. 2017;61:60–71. 28602573 10.1016/j.preteyeres.2017.06.003 PMC5653403

[34] Borysov A, Tarasenko A, Krisanova N, Pozdnyakova N, Pastukhov A, Dudarenko M, Paliienko K, Borisova T. Plastic smoke aerosol: Nano-sized particle distribution, absorption/fluorescent properties, dysregulation of oxidative processes and synaptic transmission in rat brain nerve terminals. ENVIRON POLLUT. 2020;263Pt A:114502. 10.1016/j.envpol.2020.114502 33618457

[35] Manisalidis I, Stavropoulou E, Stavropoulos A, Bezirtzoglou E. Environmental and health impacts of air pollution: A review. Front Public Health. 2020;8:14. 32154200 10.3389/fpubh.2020.00014 PMC7044178

[36] Kearney S, O’Donoghue L, Pourshahidi LK, Richardson P, Laird E, Healy M, Saunders KJ. Conjunctival ultraviolet autofluorescence area, but not intensity, is associated with myopia. CLIN EXP OPTOM. 2019;102(1):43–50. 30114725 10.1111/cxo.12825

[37] Iribarren R. Crystalline lens and refractive development. PROG RETIN EYE RES. 2015;47:86–106. 25683786 10.1016/j.preteyeres.2015.02.002

[38] Rothman KJ, Greenland S, Lash TL. Modern epidemiology. Volume 3. Wolters Kluwer Health/Lippincott Williams & Wilkins Philadelphia; 2008.

