# Supplementary material for: Long-term exposure to PM2.5 and NO2 and risk of myopia in Chinese school-aged children: a cross-sectional study
Source: BMC Ophthalmol. 2026 Jan 29;26:54. doi: 10.1186/s12886-025-04587-7 (PMC12853737; doi:10.1186/s12886-025-04587-7)
Supplement: Supplementary file 1 — Supplementary Material 1 [file 12886_2025_4587_MOESM1_ESM.docx]

| Supplementary Table S1. Subgroup analysis and effect modification for the association of myopia with PM2.5 | | | | | | | | | |
| --- | --- | --- | --- | --- | --- | --- | --- | --- | --- |
| **Subgroup** |  | **Q1** | | **Q2** | | **Q3** | | **Q4** | |
|  |  | **OR (95%CI)** | **P-interaction** | **OR (95%CI)** | **P-interaction** | **OR (95%CI)** | **P-interaction** | **OR (95%CI)** | **P-interaction** |
| Sex | Male | ref | ref | 3.10 (2.38, 4.03) | ref | 3.09 (2.13, 4.48) | ref | 3.76 (2.39, 5.90) | ref |
|  | Female | ref | ref | 4.21 (3.05, 5.81) | 0.12 | 3.62 (2.38, 5.52) | 0.03 | 3.42 (2.08, 5.63) | 0.63 |
| Grade | Kindergarten | ref | ref | 0.98 (0.50, 1.93) | ref | 0.70 (0.26, 1.86) | ref | 0.66 (0.13, 3.35) | ref |
|  | Lower Primary School (Grade 1-3) | ref | ref | 4.09 (3.16, 5.30) | 0.97 | 2.84 (1.99, 4.04) | 0.64 | 2.57 (1.63, 4.06) | < 0.001 |
|  | Upper Primary School (Grade 4-6) | ref | ref | 9.15 (4.57, 18.40) | 0.48 | 8.49 (3.93, 18.30) | 0.29 | 9.71 (4.22, 22.3) | 0.08 |
| Father education level | Below college | ref | ref | 4.23 (3.28, 5.47) | ref | 3.76 (2.64, 5.37) | ref | 4.16 (2.57, 6.75) | ref |
|  | College or above | ref | ref | 2.61 (1.79, 3.79) | 0.85 | 2.42 (1.49, 3.92) | 0.02 | 2.48 (1.49, 4.14) | 0.09 |
| Mother education level | Below college | ref | ref | 4.19 (3.21, 5.46) | ref | 3.33 (2.31, 4.79) | ref | 3.28 (2.01, 5.37) | ref |
|  | College or above | ref | ref | 2.84 (2.03, 3.97) | 0.41 | 3.27 (2.06, 5.19) | 0.05 | 3.50 (2.12, 5.80 ) | 0.15 |
| Note: The analysis was adjusted for various demographic and behavioral variables, including age, sex, maternal education, paternal education, parental myopia, pretern birth, daily outdoor activity time, daily after-school learning time, daily TV watching time. Environmental factors including average temperature and relative humidity from three years before the survey were modeled with natural cubic splines (3 degrees of freedom each). Abbreviations: PM2.5, particles with aerodynamic diameter ≤ 2.5 µm; OR, odds ratio; CI, confidence interval. | | | | | | | | | |

| **Supplementary Table S2. Subgroup analysis and effect modification for the association of myopia with NO_2_** | | | | | | | | | |
| --- | --- | --- | --- | --- | --- | --- | --- | --- | --- |
| **Subgroup** |  | **Q1** | | **Q2** | | **Q3** | | **Q4** | |
|  |  | **OR (95%CI)** | ***P*-interaction** | **OR (95%CI)** | ***P*-interaction** | **OR (95%CI)** | ***P*-interaction** | **OR (95%CI)** | ***P*-interaction** |
| Sex | Male | ref | ref | 1.95 (1.61, 2.36) | ref | 1.48 (1.14, 1.92) | ref | 1.42 (1.12, 1.79) | ref |
|  | Female | ref | ref | 1.24 (1.02, 1.52) | 0.010 | 1.14 (0.87, 1.5 ) | 0.527 | 1.12 (0.88, 1.43) | 0.145 |
| Grade | Kindergarten | ref | ref | 1.33 (0.73, 2.43) | ref | 1.27 (0.65, 2.49) | ref | 0.84 (0.45, 1.56) | ref |
|  | Lower Primary School (Grade 1-3) | ref | ref | 1.31 (1.06, 1.63) | 0.631 | 1.35 (0.99, 1.83) | 0.088 | 1.36 (1.01, 1.83) | 0.829 |
|  | Upper Primary School (Grade 4-6) | ref | ref | 1.67 (1.21, 2.32) | < 0.001 | 4.08 (2.42, 6.88) | 0.018 | 5.47 (3.4, 8.8 ) | 0.804 |
| Father education level | Below college | ref | ref | 1.19 (1.12, 1.26) | ref | 1.08 (1.00, 1.16) | ref | 1.44 (1.32, 1.57) | ref |
|  | College or above | ref | ref | 0.89 (0.82, 0.97) | 0.001 | 0.87 (0.8, 0.94) | 0.002 | 1.05 (0.95, 1.16) | 0.012 |
| Mother education level | Below college | ref | ref | 1.3 (1.09, 1.55) | ref | 1.07 (0.83, 1.4) | ref | 1.08 (0.86, 1.35) | ref |
|  | College or above | ref | ref | 2.12 (1.65, 2.73) | < 0.001 | 1.64 (1.22, 2.22) | 0.001 | 1.62 (1.23, 2.13) | 0.058 |
| Note: The analysis was adjusted for various demographic and behavioral variables, including age, sex, maternal education, paternal education, parental myopia, pretern birth, daily outdoor activity time, daily after-school learning time, daily TV watching time. Environmental factors including average temperature and relative humidity from three years before the survey were modeled with natural cubic splines (3 degrees of freedom each). Abbreviations: NO_2_, nitrogen dioxide; OR, odds ratio; CI, confidence interval; | | | | | | | | | |

| **Supplementary Table S3. Associations between air pollutants and myopia (5-year-average).** | | | |
| --- | --- | --- | --- |
| **Air Pollutants** | **Myopia** | **Left eye SE** | **Right eye SE** |
|  | **ORs and 95% CIs** | **β and 95% CIs** | **β and 95% CIs** |
| **PM2.5, μg** |  |  |  |
| per IQR increase | **1.91 ( 1.30, 2.79)** | 0.19 (-1.23, 1.61) | 0.05(-0.97, 1.06) |
| Quartile 1 (19.98-23.20) | Ref | Ref | Ref |
| Quartile 2 (23.20-36.27) | **9.85 ( 7.48, 13.0)** | -0.52 (-1.43, 0.39) | **-0.80 (-1.56, -0.04)** |
| Quartile 3 (36.27-41.04) | **11.4 (8.16, 16.0)** | -0.42 (-1.55, 0.71) | -0.67 (-1.59, 0.25) |
| Quartile 4 (41.04-56.12) | **12.7 (8.58, 18.7)** | -0.53 (-1.98, 0.92) | -0.82 (-1.9, 0.26) |
| **NO_2_, μg** |  |  |  |
| per IQR increase | 1.13 ( 0.96, 1.34) | -0.03 (-0.49, 0.43) | 0.01 (-0.32, 0.33) |
| Quartile 1 (15.88-18.25 | Ref | Ref | Ref |
| Quartile 2 (18.25-24.12) | **1.41 (1.20, 1.64)** | 0.27 (-0.35, 0.89) | -0.20 (-0.64, 0.25) |
| Quartile 3 (24.12-29.58) | **2.22 (1.74, 2.84)** | 0.04 (-0.74, 0.82) | -0.25 (-0.88, 0.37) |
| Quartile 4 (29.58-41.99) | **2.30 (1.80, 2.93)** | 0.05 (-0.72, 0.82) | -0.34 (-0.99, 0.30) |
| Note: The analysis was adjusted for various demographic and behavioral variables, including age, sex, maternal education, paternal education, parental myopia, pretern birth, daily outdoor activity time, daily after-school learning time, daily TV watching time. Environmental factors including average temperature and relative humidity from three years before the survey were modeled with natural cubic splines (3 degrees of freedom each). Abbreviations: PM_2.5_, particles with aerodynamic diameter ≤ 2.5 µm; NO_2_, nitrogen dioxide; OR, odds ratio; CI, confidence interval; IQR, interquartile range. | | | |

| **Supplementary Table S4. Associations of NO_2_ and PM_2.5_ exposure with myopia after further adjusting for O_3_.** | | | |
| --- | --- | --- | --- |
| **Air Pollutants** | **Myopia** | **Left eye SE** | **Right eye SE** |
|  | **ORs and 95% CIs** | **β and 95% CIs** | **β and 95% CIs** |
| **PM2.5, ug** |  |  |  |
| per IQR increase | **2.22 ( 1.53, 3.22)** | 0.18 (-1.23, 1.59) | 0.02 (-0.98, 1.02) |
| Quartile 1 (18.46-22.01) | Ref | Ref | Ref |
| Quartile 2 (22.01-34.49) | **3.59 (2.95, 4.38)** | -0.62 (-1.41, 0.16) | **-0.59 (-1.17, -0.01)** |
| Quartile 3 (34.49-40.48) | **3.30 (2.52, 4.32)** | -0.49 (-1.54, 0.56) | -0.38 (-1.15, 0.38) |
| Quartile 4 (40.48-54.71) | **3.49 (2.51, 4.84)** | -0.63 (-1.99, 0.74) | -0.59 (-1.57, 0.38) |
| **NO_2_, ug** |  |  |  |
| per IQR increase | 1.07 ( 0.93, 1.22) | -0.10 (-0.51, 0.31) | -0.10 (-0.40, 0.20) |
| Quartile 1 (16.09-18.34) | Ref | Ref | Ref |
| Quartile 2 (18.34-25.83) | **1.58 (1.38, 1.82)** | -0.20 (-0.79, 0.39) | **-0.50 (-0.92 , -0.09)** |
| Quartile 3 (25.83-28.41) | **1.34 (1.10, 1.62)** | -0.21(-1.05, 0.63) | -0.34 (-0.93, 0.26) |
| Quartile 4 (28.41-38.60) | **1.30 (1.09, 1.54)** | -0.30 (-1.03, 0.44) | **-**0.48 (-1.01, 0.04) |
| Note: The analysis was adjusted for various demographic and behavioral variables, including age, sex, maternal education, paternal education, parental myopia, pretern birth, daily outdoor activity time, daily after-school learning time, daily TV watching time.Environmental factors including average temperature and relative humidity from three years before the survey were modeled with natural cubic splines (3 degrees of freedom each). Abbreviations: PM_2.5_, particles with aerodynamic diameter ≤ 2.5 µm; NO_2_, nitrogen dioxide; OR, odds ratio; CI, confidence interval; IQR, interquartile range. | | | |


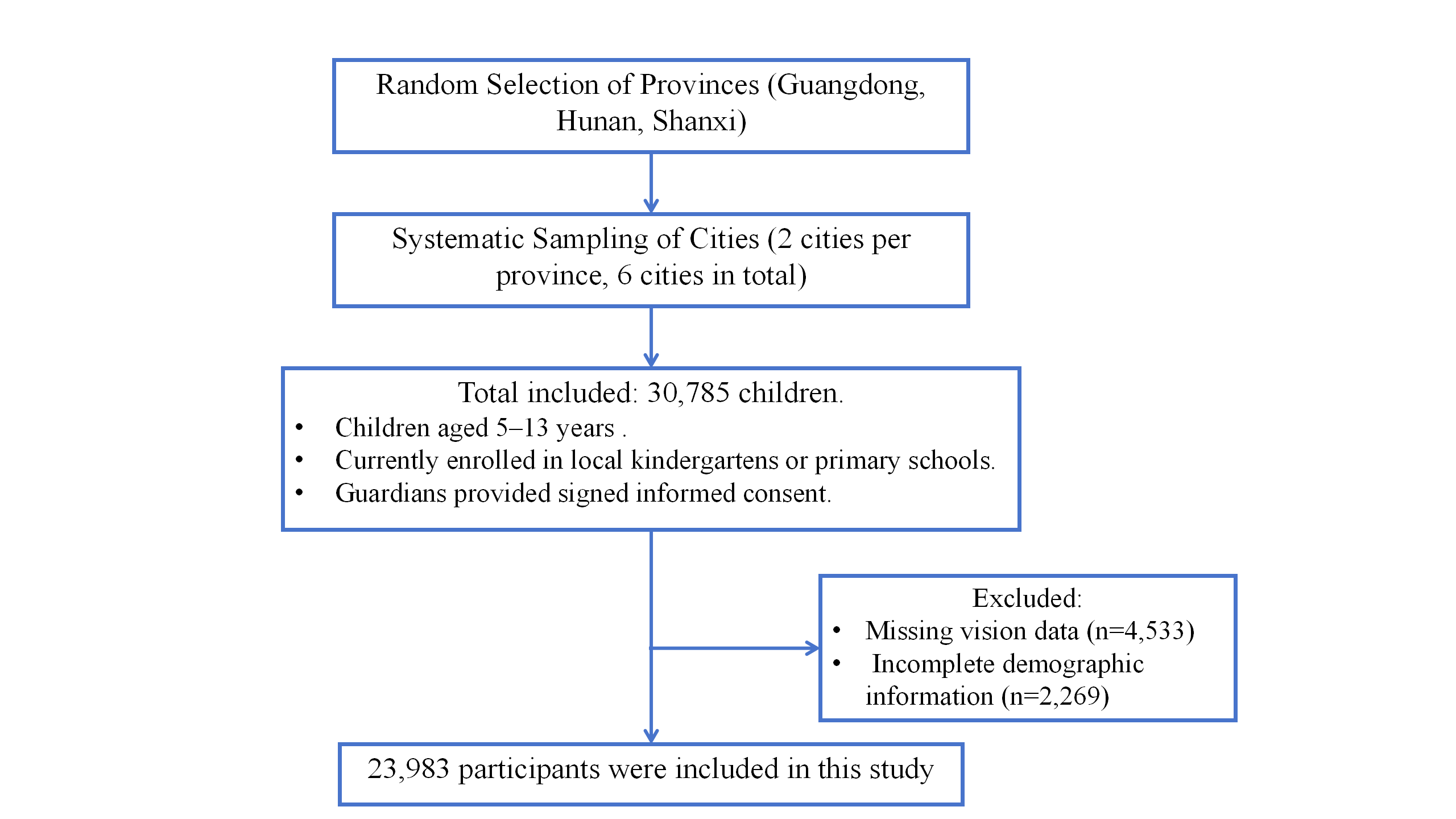


**Supplementary Figure S1. Flow diagram of study population**
